# Can Prophylactic High Flow of Humidified and Warmed Filtered Air Improve Survival from Bacterial Pneumonia and SARS-CoV-2 in Elderly Individuals? The Role of Surfactant Protein A

**DOI:** 10.3390/antiox10050640

**Published:** 2021-04-22

**Authors:** Ata Abbasi, David S. Phelps, Radhika Ravi, Joanna Floros

**Affiliations:** 1 Department of Pathology, Urmia University of Medical Sciences, Urmia 5714783734, Iran; aabbasi@alumnus.tums.ac.ir; 2 Center for Host Defense, Inflammation, and Lung Disease (CHILD) Research, Department of Pediatrics, The Pennsylvania State University College of Medicine, Hershey, PA 17033, USA; dsp4@psu.edu; 3 Division of Anesthesia, Department of Surgery, Veterans Affairs New Jersey Health Care System, 385 Tremont Avenue, East Orange, NJ 07018, USA; radhika.ravi@gmail.com; 4 Department of Obstetrics and Gynecology, The Pennsylvania State University College of Medicine, Hershey, PA 17033, USA

**Keywords:** ventilation, HFNC, COVID-19, innate immunity, surfactant protein A

## Abstract

In this opinion article, we discuss a serendipitous observation we made in a study investigating survival in aged mice after bacterial infection. This observation involved a non-invasive ventilation approach that led to variable and higher survival in male and female mice with different genetic backgrounds for the innate immune molecule, surfactant protein A (SP-A). We suggest that employing the best ventilatory modality, whether that be HFNC or another method, may augment the role of other factors such as SP-A genetics and sex in a personalized approach, and may ultimately improve the outcome.

## 1. Introduction and Discussion

Here, we discuss a serendipitous observation we made in a study investigating survival in aged mice after bacterial infection [1]. Specifically, we discuss the application of this finding to the human context and explore the potential for reducing disease severity and improving survival in individuals at risk for bacterial and viral pneumonias.

Bacterial pneumonia is a major cause of mortality and morbidity worldwide. There are various risk factors for severe disease, among which old age is one of the most important [2]. In a recent study, the incidence of hospitalization for pneumonia in patients older than 65 years was 10 times more than in younger patients [3]. Various bacterial agents are involved as etiologic factors, however Gram-positive bacteria and *Enterobacteriaceae* are the most common species [4]. *Klebsiella pneumoniae* is a Gram-negative bacterium that is colonized on human mucosal surfaces and can cause serious infections, including pneumonia and sepsis [5]. There are growing concerns about this pathogen due to various molecular changes it has undergone making it more resistant to antibiotics, and reports of severe infections caused by *K. pneumoniae* with scarce effective antibiotic therapies are on the rise [5,6,7,8]. Numerous studies have evaluated the risk factors for hypervirulent *Klebsiella* infections and explored new therapeutic approaches to improve patients’ survival and outcome [8,9,10].

Innate immunity is the non-specific first line of defense of the body, which is active in every organ against any pathogen or irritant. In the lungs, the innate immune molecule, surfactant protein A (SP-A), interacts with the sentinel cell of innate immunity, the alveolar macrophage, to modulate several processes of innate immunity. In humans (unlike in rodents), there are two SP-A genes, *SFTPA1* and *SFTPA2*, encoding SP-A1 and SP-A2, respectively; several genetic variants that may impact function have been identified for each [11,12,13,14,15]. Human surfactant proteins (SP)-A1 and SP-A2 genetic variants differentially affect several processes of the alveolar macrophage at baseline or in response to various insults [1,16,17,18,19,20,21,22,23]. They also differentially affect lung function and survival of the organism, as shown by various animal models of bacterial infection and oxidative stress [1,24,25]. These SP-A variants have been associated with differences in the susceptibility or degree of severity of various pulmonary diseases [26,27,28,29,30]. Together these observations indicate that the genetics of SP-A may play a role in innate immunity, lung function, and disease susceptibility or severity.

In our recent study [1] evaluating the correlation of SP-A variants with survival in aged mice after bacterial infection, we encountered a serendipitous finding. In this experimental model of *Klebsiella pneumoniae* infection in aged mice, we observed that mice exposed to high flow of humidified and warmed filtered air (FA) prior to infection had a better survival rate than the mice in normal room air [1]. This was the case for all humanized transgenic mice expressing different human SP-A variants, as well as in mice that lacked SP-A. However, if SP-A was present, depending on the variant and the sex of the animal, the rate of survival was significantly higher compared to those that lacked SP-A, indicating a positive impact of SP-A on survival after infection. We also observed some sex specificity with improved outcomes for the SP-A2 1A^0^ and 1A^3^ males, even when the mice were exposed to a second insult, i.e., ozone exposure. Together these findings indicate that the high-flow FA exposure under the experimental conditions used in the study not only significantly improves survival, but may also “unmask” or promote SP-A variant- and sex-specific differences implicating sex hormones and SP-A genetics, both of which have been shown previously to act in the survival of younger mice in the same experimental pneumonia model [24,31]. Among other possibilities, it was postulated that increased alveolar recruitment due to high-flow ventilation enabled the same inoculum of bacteria to spread over a larger lung surface area, resulting in a lower bacterial density in the alveolar spaces. Another possibility was that the high-flow filtered air and its consequences may have a positive impact on the host defense mechanisms by either affecting the mucociliary clearance of bacteria and innate immune functions mediated by innate immune molecules such as SP-A or by enabling a better transition from innate immunity to adaptive immunity. The importance of SP-A has been highlighted by our observation that in humans there is an association of a specific SP-A variant with better survival of lung transplant patients [28]. Therefore, it is of interest to ponder whether high-flow humidified air could further improve survival, especially during the first year when most lung failure occurs in transplant patients. Furthermore, could the utility of this treatment depend on the patient’s SP-A variants and sex?

In a recent study where the role of face masks in the hydration of the respiratory tract was investigated [32], it was observed that face masks increase the humidity of inhaled air. The authors proposed that this increased humidity is responsible for the observed decrease in SARS-CoV-2 disease severity. They further postulated that a well-humidified respiratory tract epithelium improves mucociliary clearance and maintains a well-functioning innate immune system, and that this efficacious first line of defense allows time for the adaptive immunity to come into play. This study, along with ours [1], indicates that humidified, inspired air has a positive effect on lessening disease severity, whether in bacterial or viral pneumonia. Whether high-flow, humidified, warmed air—as used in the animal studies—exerts a similarly positive effect on survival or contributes to disease severity mitigation in humans with various pulmonary diseases, especially those with either bacterial or viral infection, remains to be determined. However, it warrants further investigation because of its potential clinical relevance and impact on healthier outcomes. Of interest and relevance, humidity has been proposed as a non-pharmaceutical intervention for influenza A virus [33]. As SP-A genetics have been associated with susceptibility and severity of many pulmonary diseases in humans [26,27,28,29,30,34], it is likely that similar beneficial effects in the presence of high-flow FA may be seen in humans with pulmonary bacterial or viral infection.

Ventilation is the main therapeutic measure in patients with respiratory distress, including those with bronchiolitis [35], COPD [36], and today’s pandemic situation caused by SARS-CoV-2 infection [37]. Various non-invasive ventilation (NIV) methods have been introduced, including standard oxygenation, prone positioning, bi-level positive airway pressure (BiPAP), continuous positive airway pressure (CPAP), and high-flow nasal cannula (HFNC), among which HFNC is being widely applied in patients with respiratory distress due to its simplicity, good tolerance, safety, and also acceptable results in clinical studies [38,39]. Some studies have demonstrated its superiority over other NIV methods [40,41,42]. According to the literature, the use of HFNC has reduced the need for invasive ventilation [43,44]. Frat et al. [45] have shown that in patients with non-hypercapnic acute hypoxia only, HFNC could improve patients’ survival. It is proposed that the success of HFNC in improving the outcome of respiratory distressed patients could be due to a decrease in anatomical dead space [46,47], an improved ventilation perfusion ratio (V/Q), good tolerance due to inhalation of humidified and warm air, positive pressure generated by high nasal flow [48], and finally increased alveolar PO_2_ [45]. In contrast to other NIV methods, HFNC is the only non-invasive respiratory support that does not increase dead space and also decreases the work of breathing [38,39]. It is of interest to mention that our study [1], which led to the interesting finding (i.e., of a protective role of pre-infection of high-flow humidified and warmed air on pneumonia outcome), used a high-flow FA exposure method that shared some similarities with HNFC.

Almost all of the data in the literature about ventilation are limited to patients with respiratory failure or acute hypoxemia. This is not surprising, because without any respiratory distress there is no need for ventilatory support. Inhalation of pure or high concentrations of oxygen for a period of time may cause oxygen toxicity [49,50]. HFNC can be set up in a manner to deliver oxygen in a concentration similar to breathing room air (FiO2 = 0.21), providing enhanced ventilation of the lungs without the risk of oxygen toxicity. In light of our serendipitous finding that high-flow humidified and warmed air alleviated complications, it is reasonable to consider the potential periodic use of HFNC with an FiO2 of 0.21 in patients at high risk for the development of pulmonary infections, either bacterial or viral, and to determine whether it leads to lower infection rates and better outcomes.

The use of this non-invasive approach may be beneficial for COVID-19 patients, especially since a significant percentage of them are also infected with other pathogens [51]. However, a number of experimental and clinical studies are needed to explore the impacts of prophylactic ventilation using HNFC on lung function and on the innate immune response mediated by SP-A1 and SP-A2. The potential role of SP-A variants under different scenarios in COVID-19 patients has been discussed elsewhere [52,53].

## 2. Conclusions

In summary, our serendipitous finding provides a basic translational science foundation as a springboard towards its clinical consideration and eventual application. These unexpected observations showed that prior exposure to high-flow humidified and warmed filtered air improved survival and in certain cases improved it in an SP-A-variant- and sex-dependent manner, underscoring the roles of sex-dependent pathways and SP-A genetics in survival from bacterial pneumonia. We speculate that in addition to the beneficial effects the humidified air has on the epithelium and immune processes, especially those mediated by SP-A, high-flow ventilation, as used in the animal studies, may further facilitate recovery by enabling increased alveolar recruitment, resulting in a reduced pathogen density, better oxygenation, and reduced breathing effort. The information presented in this editorial highlights the fact that resolution of pneumonia is multifactorial and not simply dependent on ventilation. However, employing the best ventilatory modality, whether HFNC or another method, may augment the role of other factors such as SP-A genetics and sex in a personalized approach, and may ultimately improve the outcome.
